# Population Structure and Adaptive Divergence in a High Gene Flow Marine Fish: The Small Yellow Croaker (*Larimichthys polyactis*)

**DOI:** 10.1371/journal.pone.0154020

**Published:** 2016-04-21

**Authors:** Bing-Jian Liu, Bai-Dong Zhang, Dong-Xiu Xue, Tian-Xiang Gao, Jin-Xian Liu

**Affiliations:** 1 Key Laboratory of Marine Ecology and Environmental Science, Institute of Oceanology, Chinese Academy of Sciences, Qingdao, Shandong, China; 2 Laboratory for Marine Ecology and Environmental Science, Qingdao National Laboratory for Marine Science and Technology, Qingdao, China; 3 University of Chinese Academy of Sciences, Beijing, China; 4 School of Fisheries, Zhejiang Ocean University, Zhoushan, Zhejiang, China; Kunming Institute of Zoology, Chinese Academy of Sciences, CHINA

## Abstract

The spatial distribution of genetic diversity has been long considered as a key component of policy development for management and conservation of marine fishes. However, unraveling the population genetic structure of migratory fish species is challenging due to high potential for gene flow. Despite the shallow population differentiation revealed by putatively neutral loci, the higher genetic differentiation with panels of putatively adaptive loci could provide greater resolution for stock identification. Here, patterns of population differentiation of small yellow croaker (*Larimichthys polyactis*) were investigated by genotyping 15 highly polymorphic microsatellites in 337 individuals of 15 geographic populations collected from both spawning and overwintering grounds. Outlier analyses indicated that the locus Lpol03 might be under directional selection, which showed a strong homology with Grid2 gene encoding the glutamate receptor δ2 protein (GluRδ2). Based on Lpol03, two distinct clusters were identified by both STRUCTURE and PCoA analyses, suggesting that there were two overwintering aggregations of *L*. *polyactis*. A novel migration pattern was suggested for *L*. *polyactis*, which was inconsistent with results of previous studies based on historical fishing yield statistics. These results provided new perspectives on the population genetic structure and migratory routes of *L*. *polyactis*, which could have significant implications for sustainable management and utilization of this important fishery resource.

## Introduction

Marine fishes are generally expected to show much lower geographical differentiation than their freshwater counterparts, which is understandable in light of lacking geographical barriers within marine habitats as compared to freshwater habitats [1, 2]. In marine environments, many studies have failed to detect statistically significant population genetic structure among populations, which is most likely due to the persistence of extensive gene flow [1, 3, 4]. However, more and more population genetic studies based on microsatellites and SNPs have revealed low but statistically significant differentiation among populations of marine fishes, opening the possibility for detecting cryptic population structure in highly abundant and widely distributed migratory marine fishes [5–11]. Recently, studies have reported signatures of natural selection and local adaptation in marine fishes [4, 12, 13]. For example, Vandamme *et al*. [12] investigated population structure of turbot (*Scophthalmus maximus*) by using gene-associated markers in combination with seascape variables for 290 sampling locations throughout the northeast Atlantic Ocean, and results suggested that stable environmental selection pressure contributes to relatively strong local adaptation in the Baltic Sea. Larmuseau *et al*. [13] detected population differentiation in the rhodopsin gene of the sand goby (*Pomatoschistus minutus*), suggesting local adaption to water turbidity between northern and southern North Sea populations. Various environmental transitions, such as salinity and temperature gradients, have been currently demonstrated to be associated with local adaptation of populations to their native environment [12, 14]. In addition, behavioral mechanisms such as natal homing [15] as well as oceanographical features such as fronts and currents [16, 17] may also be associated with genetic divergence. Therefore, discerning contributions of different mechanisms in shaping and maintaining patterns of population differentiation as well as identifying genes under selection is essential to better understand complex population structure and dynamics of marine organisms, which is crucial for maintaining their long-term sustainability and resilience to future environmental changes [18, 19].

The small yellow croaker, *Larimichthys polyactis* (Bleeker,1877), is an important fishery species endemic to the Northwest Pacific, inhabiting coastal waters across the Bohai Sea, the Yellow Sea and the East China Sea [20]. *L*. *polyactis* is an asynchronous and multiple spawner with relatively long-distance seasonal migration [21]. Spawning grounds of *L*. *polyactis* are always located in estuaries or mixed areas of both low and high salinity near the shore area, while their overwintering grounds are located in the off shore area [22]. Overwintering aggregations of *L*. *polyactis* migrate to the spawning grounds in early April and start spawning from late April to June with a gradual spatial and temporal variation among different geographic populations [22]. After spawning, these fishes continuously aggregated into large feeding groups in nearby coastal waters, and they migrate to overwintering grounds from September to October [22, 23]. Previous studies indicated that *L*. *polyactis* were presumed to migrate towards natal sites to spawn and two geographic populations existed, one in the Northern Yellow Sea and Bohai Sea, and the other in Southern Yellow Sea and East China Sea, respectively [22–24]. As one of the most important commercial fishery resources, abundance of *L*. *polyactis* had severely declined since the 1970s in China due to anthropogenic factors such as overfishing, seawater pollution, as well as ocean current and water masses changes [25–28]. Comparing with its historical biological characteristics, distinct trends of biological parameter changes have been observed in *L*. *polyactis*, such as miniaturization, domination of low age groups, increases in growth rate but decreases in body length and weight, etc. [29]. Therefore, it is urgently needed to have a better understanding of population genetic structure and migratory routes for *L*. *polyactis*, which have important consequences for conservation, effective management and sustainable utilization of this important fishery resource.

Although *L*. *polyactis* has been the subject of many studies regarding population differentiation, studies on population dynamics, morphology, anatomy, and population genetics obtained discrepant or even conflicting results [30–34]. Previous researches based on historical fishing yield statistics could hardly provide explicit patterns of population structure as well as migration since the capture data was somehow limited [23, 24]. Meanwhile, traditional approaches of tagging experiments exhibited extremely low mark-recapture rates most likely due to the intensive fishing pressure [23, 24]. It has been previously demonstrated that oceanographic features, such as eddies and fronts, representing shift in temperature, salinity or food availability may also prevent random mixing and diffusion of marine organisms [16, 17, 35]. Although previous studies have suggested that environment factors, such as the Yellow Sea Warm Current (YSWC) and the Tsushima warm current and Kuroshio, were correlated with the migration and distribution of *L*. *polyactis*, genetic evidence for correlations between sea currents and migration or distribution of *L*. *polyactis *has not yet been reported in literature. Recent genetic studies based on RAPD and AFLP [30–32] demonstrated that there were three populations of *L*. *polyactis*, while studies based on mitochondrial (mtDNA) [33, 34] and microsatellite markers [4, 36] showed no significant population differentiation, suggesting panmixia in *L*. *polyactis*. In contrast to most previous studies, Wang *et al*. (2013) [4] found two microsatellites under diversifying selection and illustrated that the adaptive differentiation of *L*. *polyactis* could add to the understanding of the population structure and thus serves as important information for establishing conservation strategies. In addition,it is emphasized that the accuracy of genetic mixed-stock analysis (MSA) using microsatellite markers for marine fish species is mainly limited by the relatively low level of genetic divergence [37]. Therefore, a comprehensive population genetic analysis of *L*. *polyactis* is still needed to assess patterns of population genetic divergence throughout its distribution and to resolve discrepant results.

In the present study, a total of 15 novel polymorphic microsatellite markers were used to assess the levels of genetic diversity and patterns of population genetic divergence of *L*. *polyactis* populations. Our sampling scheme covered nearly the entire distribution range of *L*. *polyactis* including both nearshore spawning and offshore overwintering grounds, representing an open system experiencing multidirectional migration between relatively isolated populations across several environmental gradients. Accordingly, this sampling scheme enhanced our capability to infer processes shaping population structure and migration routes of *L*. *polyactis*. Overall, results of the present study could provide useful insights into delineating fine population genetic structure and uncovering possible processes leading to adaptive divergence in *L*. *polyactis*, which should have important implications for the management and conservation of this fishery species.

## Materials and Methods

### Ethics statement

The field studies did not involve any endangered or protected species. *L*. *polyactis* is not protected by Chinese law or by any of the countries where and when sampling was performed. It is a commercially harvested species in China and the other Northeast Asian countries. The samples were collected by trawling and fishes were already dead when collected.

### Sampling and DNA extraction

**Fig 1:**
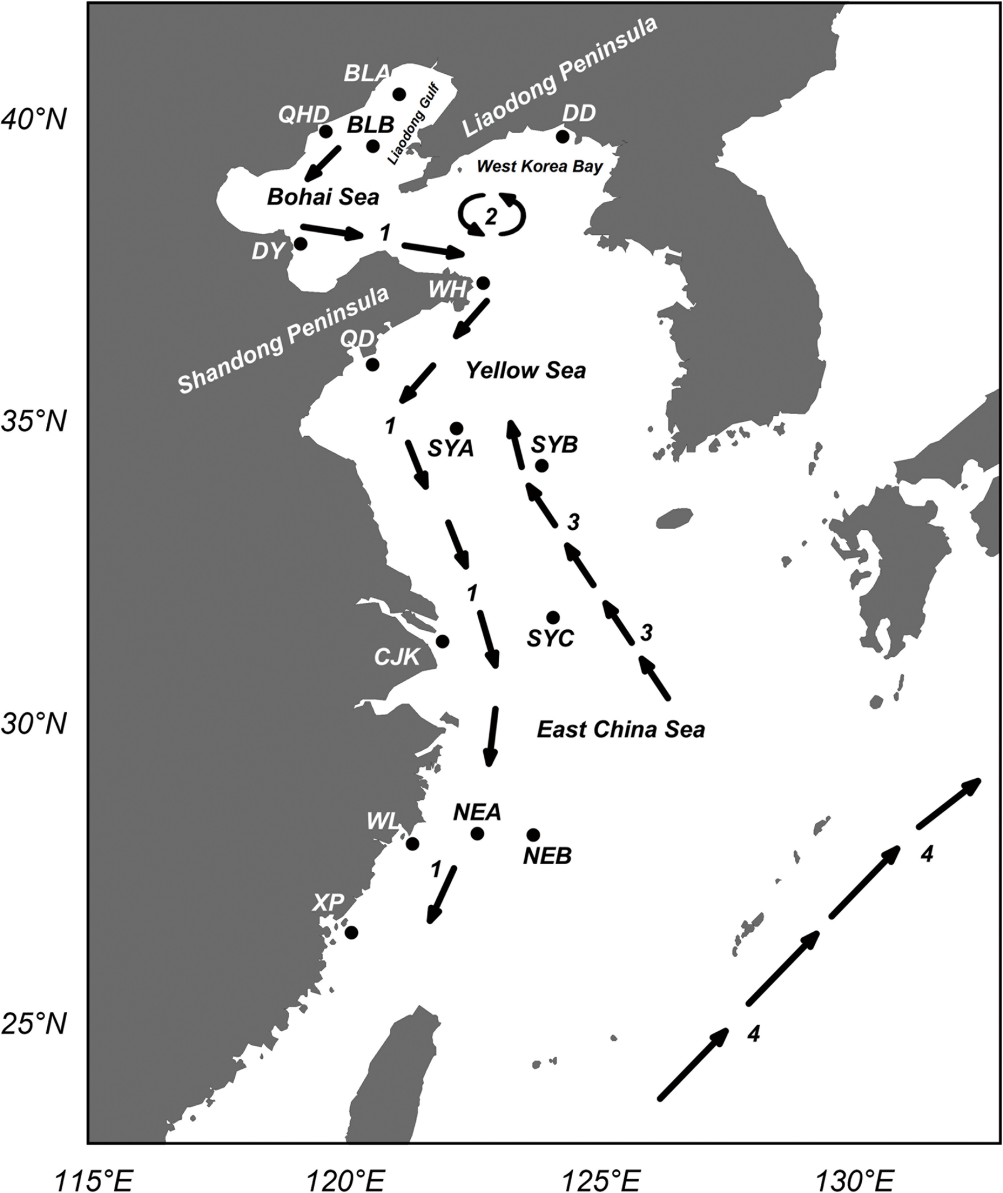
Map showing sample locations of *L*. *polyactis* and ocean currents (1 to 4). Detailed sample information is shown in Table 1. 1, China Coastal Current; 2, Northern Yellow Sea Cold Water Mass (NYSCWM); 3, Yellow Sea Warm Current (YSWC); 4, Kuroshio Current.

A total of 337 *L*. *polyactis* adult individuals were collected from 15 geographical locations (Fig 1; Table 1), including 10 locations along the coast of China (Dandong, BLA, BLB, Qinhuangdao, Dongying, Weihai, Qingdao, Wenling, Xiapu and Yangtze River Estuary) and five locations from offshore areas of the Yellow Sea and the East China Sea (SYA, SYB, SYC, NEA, NEB). All the samples were collected from natural fishing grounds between March 2005 and September 2014 using direct fishing techniques. Caudal fin or muscle tissue samples were collected and preserved in 95% (v/v) ethanol at -80°C. Total genomic DNA was extracted using the standard phenol-chloroform protocol. Extracted DNA was checked using 1.5% agarose gel electrophoresis and then stored at -20°C.

**Table 1:** Sampling information and genetic variation indices for microsatellites.

Locality	Abb.	Coordinates	Sampling date	Sample size	Av *H*_O_	Av *H*_E_	Av *PIC*	Av *A*_R_	*F*_IS_
Dandong	DD	39°50′N,124°12′E	2014.05	24	0.806	0.888	0.857	11.677	0.095
BLA	BLA	40°15′N,121°45′E	2007.10	24	0.794	0.886	0.855	11.233	0.106
BLB	BLB	39°45′N,120°45′E	2007.10	24	0.803	0.890	0.860	11.188	0.100
Qinhuangdao	QHD	39°55′N,119°37′E	2014.09	15	0.822	0.873	0.828	10.942	0.060
Dongying	DY	37°45′N,119°15′E	2009.09	16	0.687	0.862	0.814	10.085	0.209
Weihai	WH	37°20′N,123°20′E	2009.09	24	0.770	0.889	0.858	11.317	0.136
Qingdao	QD	36°N,120°30′E	2009.09	20	0.796	0.876	0.840	10.933	0.094
SYA	SYA	35°19′N,122°E	2005.04	24	0.767	0.891	0.860	11.137	0.142
SYB	SYB	33°58′N,123°30′E	2005.04	24	0.735	0.881	0.848	10.815	0.168
SYC	SYC	31°45′N,124°05′E	2005.03	23	0.793	0.895	0.864	11.300	0.116
Yangtze River estuary	CJK	31°05′N,121°59′E	2013.11	20	0.797	0.875	0.839	11.191	0.092
NEA	NEA	28°30′N,123°30′E	2007.09	23	0.823	0.900	0.869	11.824	0.087
NEB	NEB	28°30′N,123°E	2007.09	30	0.717	0.887	0.860	11.111	0.194
Wenling	WL	28°15′N,121°29′E	2014.03	22	0.827	0.889	0.857	11.463	0.072
Xiapu	XP	26°50′N,120°07′E	2014.03	24	0.783	0.887	0.856	11.515	0.119

Abbreviation of populations (Abb.), average of observed heterozygosity (Av *H*_O_), average of expected heterozygosity (Av *H*_E_), average of Polymorphism Information Content (Av *PIC*), average of allelic richness (Av *A*_R_) and inbreeding coefficient (*F*_IS_).

### Microsatellite Genotyping

All samples were genotyped at 15 microsatellite loci (Lpol02, Lpol03, Lpol04, Lpol05, Lpol06, Lpol08, Lpol09, Lpol10, Lpol11, Lpol12, Lpol13, Lpol14, Lpol15, Lpol16 and Lpol17) originally developed by Liu *et al*. [38]. The 15 highly polymorphic loci were amplified following the PCR protocol described in [39]. Fluorescently-labeled PCR products were electrophoresed on an ABI3730 XL DNA sequencer with the LIZ-500 size standard (Applied Biosystems). Alleles were called using GENEMAKER software version 2.2.0 (Soft-Genetics, State College, PA, USA). The microsatellite genotype data for *Larimichthys polyactis* is provided as a supporting information file (S1 File).

### Data analysis

#### Genetic diversity

For each population, genetic variation indices including polymorphism information content (*PIC*), observed heterozygosity (*H*_O_) and expected heterozygosity (*H*_E_) were calculated using the Excel Microsatellite Toolkit [40]. Inbreeding coefficient (*F*_IS,_ a measure of the extent of nonrandom mating), and allelic richness (*A*_R,_ an estimate of allelic diversity independent of sample size) were computed in FSTAT 2.9 [41]. Deviations from Hardy-Weinberg equilibrium (HWE) for each locus in each population and linkage disequilibrium (LD) between pairs of loci were tested in GENEPOP 4.3 using the Markov chain methods (10000 dememorizations, 1000 batches, and 10000 iterations per batch) [42, 43]. The software MICRO-CHECKER 2.2.0 [44] was used to test for technical artefacts such as null alleles, stuttering and large allele dropout.

#### Outlier tests

FIDST2 [45] implemented in LOSITAN [46] was applied to identify potential outliers. This method calculates *F*_ST_ for each locus and assumes that outlier loci under diversifying selection would show increased level of population differentiation compared to simulated distribution of loci based on observed level of population differentiation. Thus loci with unusually high *F*_ST_ values were regarded as potentially being under directional selection. LOSITAN was run using 100 000 simulations with a confidence intervals of 99.5%, a false discovery rate (FDR) of 0.05, and assuming a stepwise mutation model. Moreover, it has been demonstrated that the results based on LOSITAN may be biased by the presence of strong hierarchical population structure leading to increased level of false positives [47]. Therefore, we further performed the test based on the method of Beaumont & Nichols [45] as described by Excoffier *et al*. [47], which was performed under a hierarchical island model with 100 000 simulated loci using Arlequin 3.5 [48].

#### Population structure and differentiation

Pairwise *F*_ST_ values were estimated based on neutral microsatellite dataset and locus Lpol03 and significance was adjusted using the Benjamini–Yekutieli multiple testing correction [49].The global *F*_ST_ was calculated by GENEPOP 4.3. In order to compare the patterns of genetic structure between neutral and possible selected loci, principal coordinate analysis (PCoA) based on gene frequency data was performed using GenAlEx 6.5 [50]. The software STRUCTURE 2.3 [51, 52] was applied to cluster the samples on the basis of their microsatellite genotypes. We performed an independent assessment of the effects of both neutral and outlier loci on genetic structure. Ten replicates were run for all possible values of the maximum number of clusters (K) up to K = 16 using the admixture model, and for each run, 2,000,000 iterations were carried out after a burn-in period of 200,000 iterations. The Evanno’s method [51] implemented in Structure Harvester website was used to detect the optimum number of genetically homogeneous groups (K) [53]. In order to test the partitioning of genetic variation within and among putative groupings of samples, a hierarchical analysis of molecular variance (AMOVA) was conducted based on neutral microsatellite dataset with 10000 permutations using Arlequin 3.5.

#### Demographic history and isolation by geographical distance

Bottleneck software ver. 1.2.02 [54] was used to verify the existence of bottlenecks with 1,000 iterations under the infinite allele model (IAM), stepwise-mutation model (SMM) and two-phased model of mutation (TPM). Significance was tested using the Wilcoxon signed-rank test (Wilcoxon, 1945). Qualitative test of model shift was performed to calcuate the allele frequency distribution using Bottleneck 1.2.02 [54]. The effects of geographical isolation on the genetic structure were assessed by the pattern of isolation by distance (IBD). Pairwise geographic distance between two collections was estimated as the nearest marine distance using Google Maps. IBD regression analysis was performed online using the IBD web service [55] with 10,000 randomizations.

## Results

### Genetic diversity

Summary statistics of 15 microsatellite loci in the 15 populations are shown in Table 1 & S1 Table. A total of 462 alleles were detected across all 15 loci, with the number of alleles per locus ranging from 15 for locus Lpol11 to 43 for locus Lpol15. Average *A*_R_ per sample was highest in NEA (11.824) and lowest in DY (10.085). The average of expected heterozygosity ranged from 0.862 (DY) to 0.900 (NEA) and the average of observed heterozygosity varied from 0.870 (DY) to 0.924 (CJK), respectively. The average of *PIC* values ranged from 0.814 to 0.869 (Table 1), suggesting a high level of genetic diversity. For all samples and loci combinations, no linkage disequilibrium was detected in the 15 populations. However, seven cases of locus-population combination out of 240 showed significant departure from Hardy-Weinberg equilibrium (HWE) after sequential Bonferroni correction (*P* < 0.0005, S1 Table). The MICRO-CHECKER analysis revealed that the loci Lpol05, Lpol11, Lpol16 and Lpol17 may include null alleles in one to four populations. However, we used all loci in this study because no locus was affected by null alleles in all samples.

### Outlier detection

Two loci (Lpol03 and Lpol04) were identified as outliers at the 99.5% confidence interval after FDR correction, with *F*_ST_ values of 0.094 and 0.023, respectively (S1 Fig). Nevertheless, there were also two loci (Lpol03 and Lpol06) identified as putatively under positive selection at the significant level of 0.01 under hierarchical island model (S2 Fig). The consistent results of both outlier tests suggested Lpol03 was a candidate locus under directional selection. The result of NCBI blastn search against refseq_genomic database for the flanking region sequences of Lpol03 showed strong similarity to Grid2 gene (*E*-value = 0; Identity = 97%) which encodes the glutamate receptor δ2 protein (GluRδ2), an excitatory receptor for glutamate. The Lpol03 with a fragment of 454 bp was found to be located in the intron of Grid2 gene in a genomic scaffold of *Larimichthys crocea*.

### Genetic differentiation and population structure

**Fig 2:**
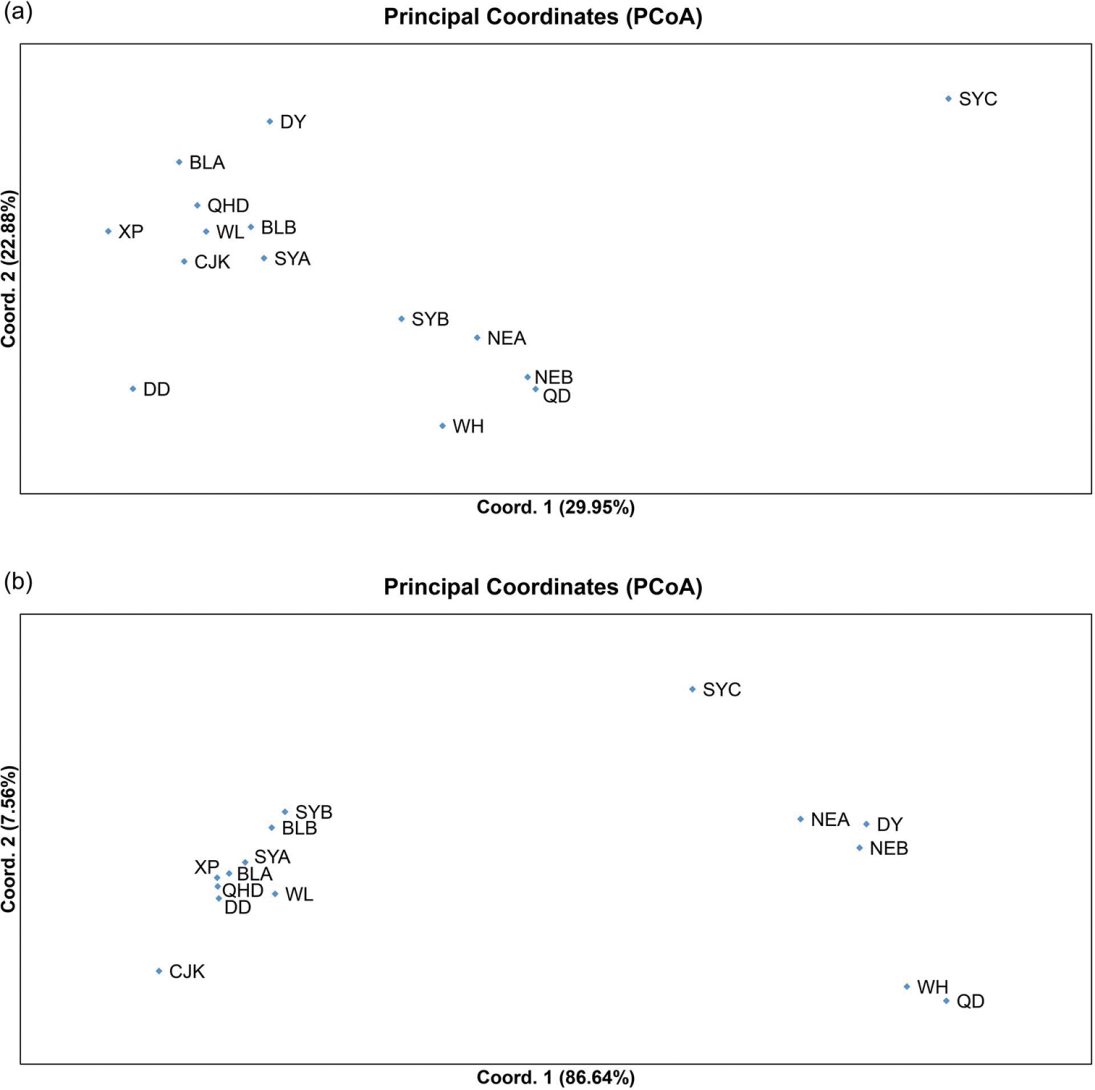
PCoA plotting of population differentiation based on (a) neutral microsatellites; (b) locus Lpol03.

**Fig 3:**
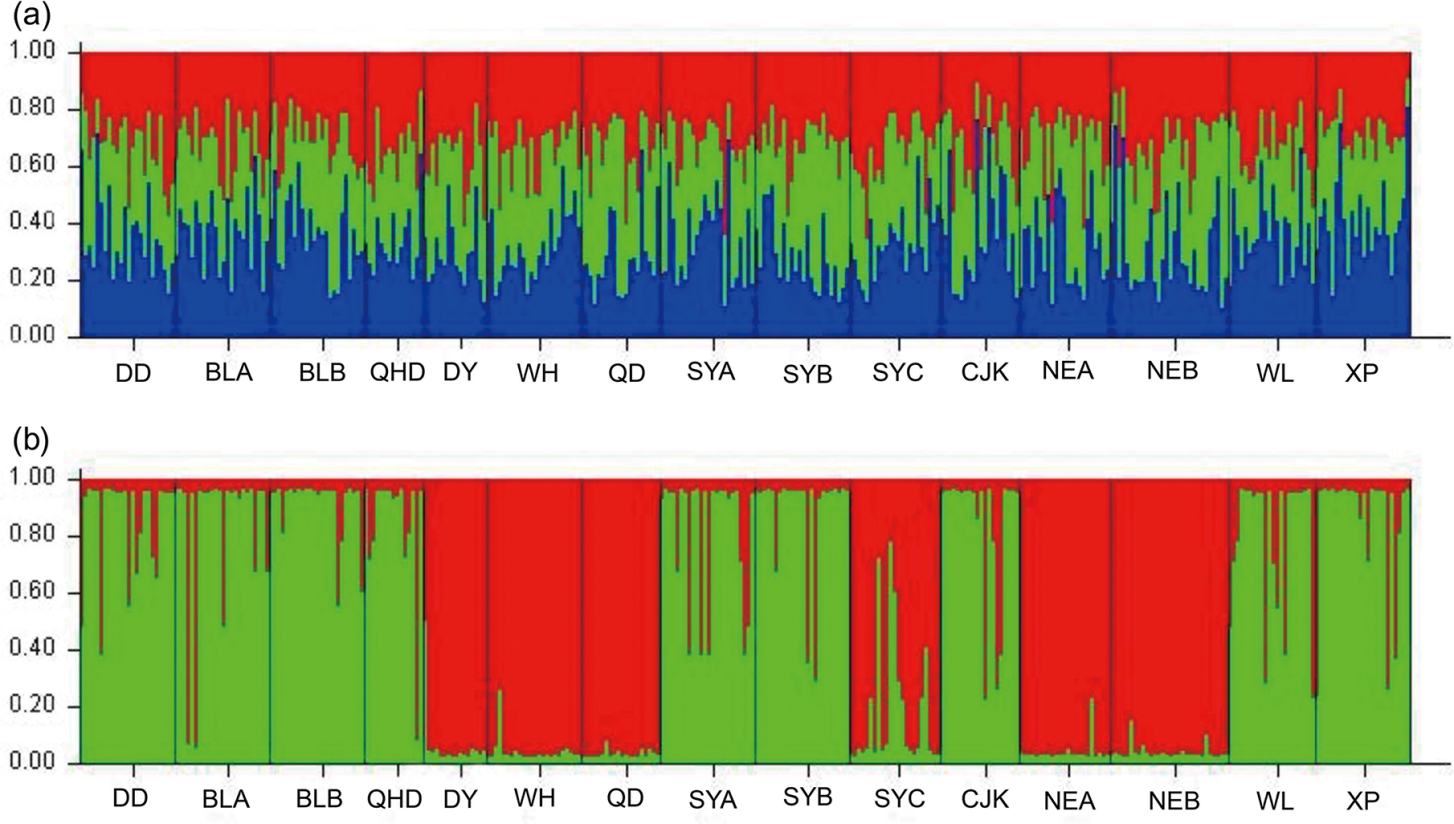
**Plotting results of the program STRUCTURE for neutral loci (a) with K = 3, and for locus Lpol03 (b) with K = 2, respectively.** Each population is shown as plots and each individual is represented by a single vertical line of different colors, indicating the groups they belong to.

The global *F*_ST_ among all populations based on neutral loci and the outlier locus Lpol03 were 0.0036 and 0.0915, respectively. Pairwise *F*_ST_ values among samples based on neutral loci ranged from -0.0084 (between NEA and NEB) to 0.0184 (between SYC and CJK). Moreover, most *F*_ST_ values between SYC and other sampling populations were statistically significant after Benjamini–Yekutieli correction (Table 2). Approximately half the comparisons based on locus Lpol03 yielded highly significant *F*_ST_ values after Benjamini–Yekutieli correction (ranging from 0.0772 to 0.2543, *P* < 0.00908). Pairwise *F*_ST_ results based on the outlier locus Lpol03 exhibited an overall higher value than those based on neutral loci, suggesting the existence of genetically differentiated populations of *L*. *polyactis* under directional selection. PCoA plotting based on neutral loci indicated no clear population structure among populations and a genetic distinctiveness of sample SYC (Fig 2A). However, PCoA plotting for locus Lpol03 showed two clearly differentiated groups: DD,BLA,BLB,QHD,SYA,SYB,CJK,WL and XP clustered as one group, while QD,WH,DY, NEA, NEB and SYC grouped as another distinct cluster (Fig 2B). Overall, the PCoA plotting analysis illustrated a pattern consistent with previous estimates of population structure derived from pairwise *F*_ST_ based on the neutral loci and outlier locus Lpol03. In simulations of the Bayesian clustering method to evaluate genetic structure, the mean ΔK values suggested 3 clusters as the most likely population structure for the neutral dataset (S3 Fig), while the number of genetic groups best fitting for the outlier locus Lpol03 was 2 (S4 Fig). Using *K* = 3 in STRUCTURE, we found that the 15 populations did not show any distinct population genetic structure based on the neutral datasets. Conversely, strikingly different result was obtained based on Lpol03 locus, which showed that two distinct genetic clusters were identified (Fig 3). When the AMOVA was performed without considering hypothetical grouping of populations, no significant genetic variability was observed among populations. However, the hierarchical AMOVA conducted with populations grouped according to sampling time revealed low but significant genetic variance among groups (*P* = 0.000) respectively. Conversely, with populations grouped according to geographic origins, the AMOVA showed nonsignificant genetic differentiation among groups (*P* = 0.112 and 0.131) respectively (S2 Table).

**Table 2:** Matrix of pairwise *F*_ST_ values between populations based on neutral microsatellite datasets (below diagonal) and locus Lpol03(above diagonal).

	DD	BLA	BLB	QHD	DY	WH	QD	SYA	SYB	SYC	CJK	NEA	NEB	WL	XP
DD		-0.0140	0.0028	-0.0078	**0.1769**	**0.1902**	**0.2170**	0.0019	0.0179	**0.1158**	-0.0076	**0.1359**	**0.1594**	-0.0104	-0.0112
BLA	0.0060		-0.0052	-0.0072	**0.1687**	**0.1857**	**0.2137**	-0.0015	0.0085	**0.1083**	-0.0010	**0.1314**	**0.1512**	-0.0053	-0.0113
BLB	0.0054	-0.0019		-0.0014	**0.1519**	**0.1689**	**0.1919**	0.0012	-0.0133	**0.0889**	0.0267	**0.1131**	**0.1390**	0.0032	-0.0083
QHD	0.0022	0.0026	0.0038		**0.1773**	**0.1930**	**0.2175**	-0.0056	0.0261	**0.1056**	-0.0051	**0.1354**	**0.1641**	-0.0161	-0.0145
DY	0.0037	-0.0002	0.0059	0.0042		0.0076	0.0209	**0.1692**	**0.1564**	0.0195	**0.2208**	0.0088	-0.0078	**0.1511**	**0.1745**
WH	0.0020	0.0074	0.0054	0.0060	0.0080		-0.0140	**0.1848**	**0.1746**	**0.0772**	**0.2265**	0.0223	0.0066	**0.1649**	**0.1905**
QD	0.0005	0.0063	0.0067	0.0070	0.0020	0.0006		**0.2139**	**0.1980**	**0.0910**	**0.2543**	0.0306	0.0219	**0.1884**	**0.2148**
SYA	0.0019	0.0027	0.0053	0.0063	0.0044	0.0044	0.0086		0.0194	**0.1102**	0.0199	**0.1285**	**0.1496**	0.0038	-0.0019
SYB	0.0078	0.0054	0.0015	0.0068	0.0131	0.0090	0.0020	0.0089		**0.0984**	0.0454	**0.1187**	**0.1432**	0.0250	0.0065
SYC	**0.0192**	**0.0127**	**0.0121**	**0.0119**	0.0122	**0.0130**	0.0096	**0.0120**	**0.0124**		**0.1613**	0.0166	0.0322	**0.0917**	**0.1098**
CJK	0.0043	0.0088	0.0068	0.0081	0.0029	**0.0114**	0.0097	0.0098	0.0094	**0.0184**		**0.1763**	**0.2000**	0.0030	-0.0051
NEA	0.0029	0.0025	0.0038	0.0041	0.0030	0.0016	0.0011	0.0006	0.0035	0.0046	0.0045		-0.0053	**0.1129**	**0.1353**
NEB	0.0061	0.0066	0.0052	0.0072	0.0067	0.0028	0.0019	0.0063	0.0054	**0.0100**	0.0080	-0.0084		**0.1378**	**0.1629**
WL	0.0010	0.0080	0.0048	0.0027	0.0069	0.0076	**0.0108**	0.0026	**0.0117**	**0.0128**	0.0066	0.0031	0.0092		-0.0050
XP	0.0026	0.0052	0.0031	0.0006	0.0042	0.0086	0.0054	0.0069	0.0072	**0.0161**	0.0056	0.0043	0.0067	0.0035	

Significant values after a Benjamini–Yekutieli correction based on the false discovery rate approach (*P* < 0.00908) are highlighted in bold.

### Population demography and isolation by distance

Genetic bottleneck analysis showed no significant heterozygotes excess in all of the 15 populations under the two-phase model (TPM) and the stepwise mutation model (SMM), only the test based on the infinite alleles model (IAM) identified several excess of heterozygosity (S3 Table). Given that microsatellite mutations are thought to occur largely through a stepwise process, a combination of the SMM and IAM is expected to provide best estimate of equilibrium heterozygosity for the bottleneck analysis [56]. The absence of heterozygosity excess under both the SMM and the TPM models suggested that all the populations were at mutation-drift equilibrium. These results were also consistent with the normal L-shaped distribution of allele frequency, indicating no genetic bottleneck in the recent past. IBD analysis showed that there was no evidence of isolation by distance pattern (*P* > 0.05), potentially indicating the persistence of high-level gene flow.

## Discussion

### Genetic diversity and population differentiation

In the present study, slight heterozygote deficiency in all populations was observed, which might be attributed to Wahlund effect resulting from mixing of fish from different breeding areas [57]. Deviation from HWE might be explained by decreases in population size as a result of commercial exploitation, in which the consequences are similar to those of the founder effect [58, 59]. In addition to population-level processes, the null alleles frequently found at microsatellite loci, which also have been reported in many other fish species [60–62], could probably explain deviations from HWE. The high genetic diversity in microsatellite loci of *L*. *polyactis* might be attributed to high mutation rate and large population size [63]. The 15 highly polymorphic microsatellites provided much more genetic information than the RAPD, AFLP and mtDNA markers in previous studies on *L*. *polyactis* [30, 31, 34, 64, 65]. Moreover, as suggested by AMOVA and IBD regression analysis, no evidence of isolation by distance was observed, which further reflected high degree of genetic connectivity among different geographical populations of *L*. *polyactis*. However, it should be noted that differentiation between groups clustered by sampling time was supported by the AMOVA (S2 Table), even though the proportion of genetic variation is relatively low.

### Combining neutral and selected loci to assess population structure

It has been suggested that genomic regions showing high divergence may represent signatures of local adaptation or genomic islands of divergence recalcitrant to gene flow [66, 67]. In the present study, no significant population structure was identified in *L*. *polyactis* based on neutral microsatellite markers, suggesting the existence of high gene flow, which was consistent with previous results based on microsatellite markers and mtDNA analysis [34, 36]. Conversely, strikingly different results were obtained when using Lpol03 locus identified as candidate outlier potentially under positive selection, which showed evidence for predominant divergence among populations of *L*. *polyactis*. Significant population differentiation were also observed based on outlier microsatellite loci associated with temperature in Wang *et al*. (2013). No isolation by distance was detected among populations of *L*. *polyactis*, which was also in accordance with previous results using both neutral and outlier microsatellite loci in Wang *et al*. [4]. The patterns of population divergence observed in the present study were generallyconsistent with previous studies based on a different battery of microsatellites, reflecting the effects of both gene flow and local adaptation on population structure.

The genomic sequence containing the outlier Lpol03 showed a strong homology with Grid2 gene encoding the glutamate receptor δ2 protein (GluRδ2), an excitatory receptor for glutamate, which is critically important for dendrite development and synapse formation during neuronal growth such as motor learning and neural wiring [68, 69]. Mikami *et al*. found that the expression of zebrafish GluRδ2 is selective for cerebellum-like neural wiring [69]. In general, glutamate neurotransmitters are involved in a variety of stimulus-induced actions such as stress, aggressiveness, locomotion as well as exploring behavior, which were considered to be important for food accessibility and size-related sex determination for fish [70]. As most anadromous fishes have evolved strict homing behavior, significant variation in motor learning behavior may also be attributed to separate wild groups matched to different strict homing behaviors with spatial and temporal changes [71]. It has been demonstrated that false positives of outlier detection can result from recent population bottlenecks or geographical structure [72, 73]. Neither genetic bottleneck nor isolation by distance was detected in *L*. *polyactis*, providing support to the validation of the outlier loci detected. Therefore, these results led us to conclude that a cryptic adaptive population structure might exist in populations of *L*. *polyactis*, hence illustrating that local selection pressures might override homogenizing effects of high level gene flow [14, 74].

### Evidence for isolation by marine environment in overwintering aggregations

In the present study, all the analyses based on the outlier locus Lpol03 indicated that two distinct genetically distinct groups existed among the 15 samples. Moreover, clear genetic differentiation were detected between the overwintering samples from the Yellow Sea (SYA, SYB) and those from the East China Sea (NEA, NEB), indicating that there were two genetically distinct overwintering aggregations, which was consistent with the previous findings based on capture fishery data [23]. During the winter monsoon from late October to early April, a period when *L*. *polyactis* aggregate in the overwintering grounds, a strong thermohaline front forms in the southeastern Yellow Sea (YSWC), which represents an area with a sharp cline in oceanographical conditions such as temperature and salinity and thus acts as physical barriers to exchange in the marine environment [75]. Likewise, environmental mechanisms, as contributing and interacting factors in determining population distribution of marine fish, have continually drawn attention in recent population dynamics studies of marine organisms [76]. For example, Li *et al*. [77] found that the wintering migration of Japanese anchovy (*Engraulis japonicus*) could be driven by spatially varying environmental factors such as water temperature and salinity, and both Yellow Sea Cold Water Mass (YSCWM) and YSWC were demonstrated to have a remarkable influence on the migration and distribution of Japanese anchovy. Moreover, genetic composition of individuals from the overwintering population of SYC were more affiliated with those from the East China Sea overwintering populations (NEA, NEB). Xu *et al*. (2009) [24] reported that the overwintering aggregations in the southern Yellow Sea are admixtures of relatively isolated spawning groups of *L*. *polyactis*, which is consistent with findings in the present study. Possible causes for this process may be attributed to the fact that the YSWC weakens in late spring when the spawning migration of *L*. *polyactis* begins [75, 78], which therefore leads to a temporal exchange of individuals from the two isolated overwintering aggregations in the Yellow Sea and East China Sea.

### Inference for novel migration routes of *L*. *polyactis*

Population structure analyses based on the outlier locus Lpol03 indicated that spawning individuals from WL and XP showed more similarity to the Yellow Sea overwintering population (SYA and SYB) rather than the East China Sea overwintering group (NEA and NEB). The result was consistent with previous results detected by diversifying microsatellite markers, which indicated that populations in the CECS (Central East China Sea) were significantly differentiated from those in the NECS (North East China Sea) [4]. In addition, no clear geographical pattern of population genetic differentiation was observed in the present study based on the outlier locus Lpol03, which is consistent with the population structure detected with loci under diversifying selection in Wang *et al*. [4]. Therefore, our results suggested that spawning groups of *L*. *polyactis* inhabiting in the two overwintering grounds were locally adapted to multiple migration routes. For the Yellow Sea overwintering aggregation: one spawning group migrated northward to the northern part of Bohai Sea and Yellow Sea; the other one migrated southward to the coastal waters of the Central East China Sea and the Yangtze River estuary. Furthermore, overwintering aggregations from the North East China Sea mainly migrated to coastal areas off Shandong Peninsula. Interestingly, individuals of both overwintering groups spawn in separate areas of the Bohai Sea (Fig 1). One important oceanographic factor influencing the migration and distribution of *L*. *polyactis* in the Bohai Sea could be the existence of Northern Yellow Sea Cold Water Mass (NYSCWM), which is a unique hydrographic phenomenon in the Yellow Sea [79]. The center of NYSCWM locates outside Bohai Strait, forming a stable water mass with low temperature and high salinity from May to October, which may affect the spawning and overwintering migration routes of *L*. *polyactis* between the two overwintering grounds in the Yellow Sea and the East China Sea and the coastal spawning areas [79].

## Conclusions

Although high gene flow was indicated by the neutral microsatellites, two genetically differentiated overwintering populations of *L*. *polyactis* were highlighted by the outlier locus potentially under selection, one mainly in the East China Sea and the other in the Yellow Sea. Accordingly, a novel migration pattern of *L*. *polyactis* was also suggested, which was inconsistent with results of previous studies inferred by traditional approaches [23]. It should be noted that although potential migrants were identified based on the outlier data, suggesting the existence of gene flow between the two migratory groups of *L*. *polyactis*, there were also strong evidence that gene flow between the two migratory groups might be limited by local adaptation. Population structure based on the outlier locus Lpol03 reported in our study exhibited a high level of genetic differentiation, which was in concordance with previous results based on genetic markers as well as capture data. In total, the consistent results suggested that the outlier locus Lpol03 was a candidate loci with stronger discriminating power leading to increased accuracy for future MSA studies. Future research should aim to generate a comprehensive link between the spawning groups and overwintering aggregations of *L*. *polyactis*, and to use the gene-associated marker in combination with seascape variables, which would provide a powerful means for uncovering the processes leading to adaptive divergence. Overall, the present study provided new insights into the population genetic structure and migratory routine of *L*. *polyactis*, which would have significant implications for the sustainable management and utilization of this important marine fishery resource.

## Supporting Information

S1 Fig Comparison of *F*_ST_ and *H*_E_ in the polymorphic loci of *L*. *polyactis* used to identify outliers and potential candidates for selection with the LOSITAN software. The graphical output shows the simulated confidence area for neutral loci (*pale gray*), positive selection (*red*) and balancing selection (*yellow*). Loci outliers are tagged with labels.
(TIF)

S2 Fig Graphical output of the joint distribution of heterozygosity (*H*_E_) and *F*_ST_ generated by ARLEQUIN to detect loci potentially under selection in a hierarchical level. Loci significant at the 5% level are shown as *filled blue circles*, while loci significant at the 1% level are shown as *red filled circles*. Loci below or above the *red lines* are marked as *ﬁlled red circles* and correspond to markers potentially under balancing or directional selection, respectively.
(TIF)

S3 Fig ΔK values calculated according to Evanno et al. (2005) for neutral loci data sets. (TIF)

S4 Fig ΔK values calculated according to Evanno et al. (2005) for outlier locus Lpol03. (TIF)

S1 File Microsatellite genotype data for *Larimichthys polyactis*. (TXT)

S1 Table Summary of the statistics for fifteen microsatellite loci. (DOCX)

S2 Table Analysis of molecular variance (AMOVA) based on neutral microsatellite datasets. (DOCX)

S3 Table Signed rank Wilcoxon test of the mutation–drift equilibrium estimated for fifteen microsatellite loci in populations of *L*. *polyactis*. (DOCX)
