# Supplementary figures and images for: Population Structure and Adaptive Divergence in a High Gene Flow Marine Fish: The Small Yellow Croaker (Larimichthys polyactis)
Source: PLoS One. 2016 Apr 21;11(4):e0154020. doi: 10.1371/journal.pone.0154020 (PMC4839715; doi:10.1371/journal.pone.0154020)

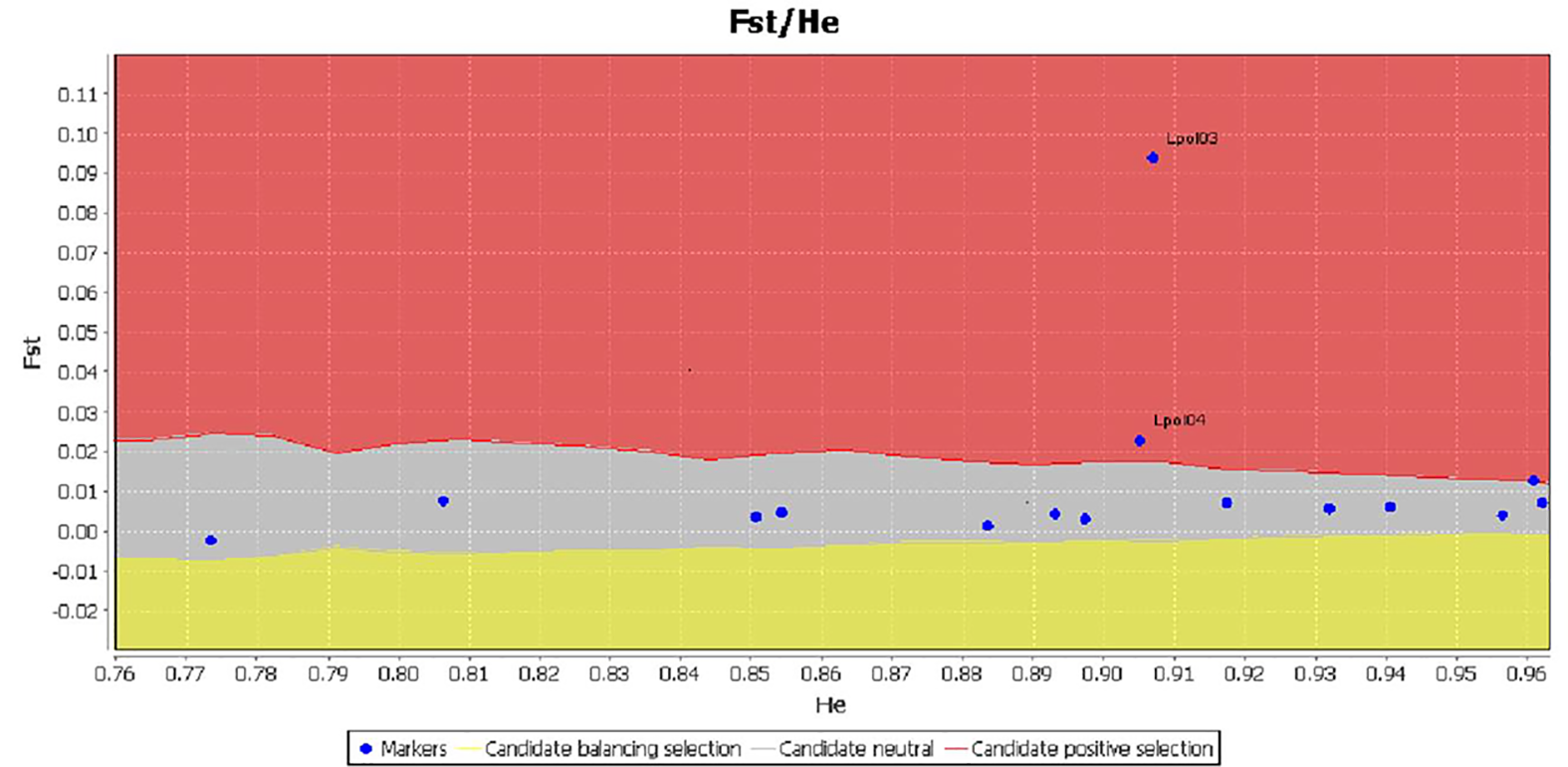

Supplement: S1 Fig — The graphical output shows the simulated confidence area for neutral loci (pale gray), positive selection (red) and balancing selection (yellow). Loci outliers are tagged with labels. (TIF) [file pone.0154020.s001.tif]

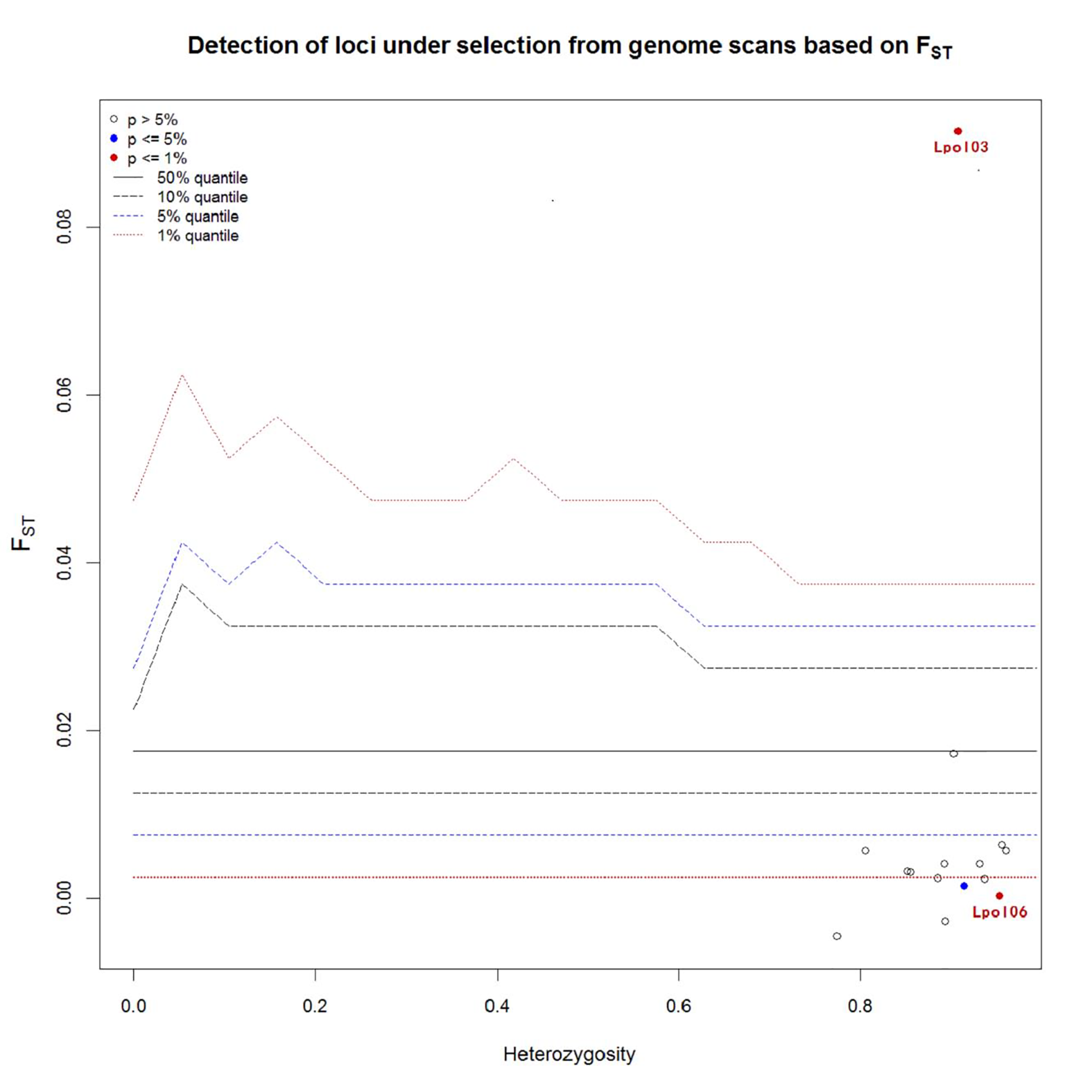

Supplement: S2 Fig — Loci significant at the 5% level are shown as filled blue circles, while loci significant at the 1% level are shown as red filled circles. Loci below or above the red lines are marked as ﬁlled red circles and correspond to markers potentially under balancing or directional selection, respectively. (TIF) [file pone.0154020.s002.tif]

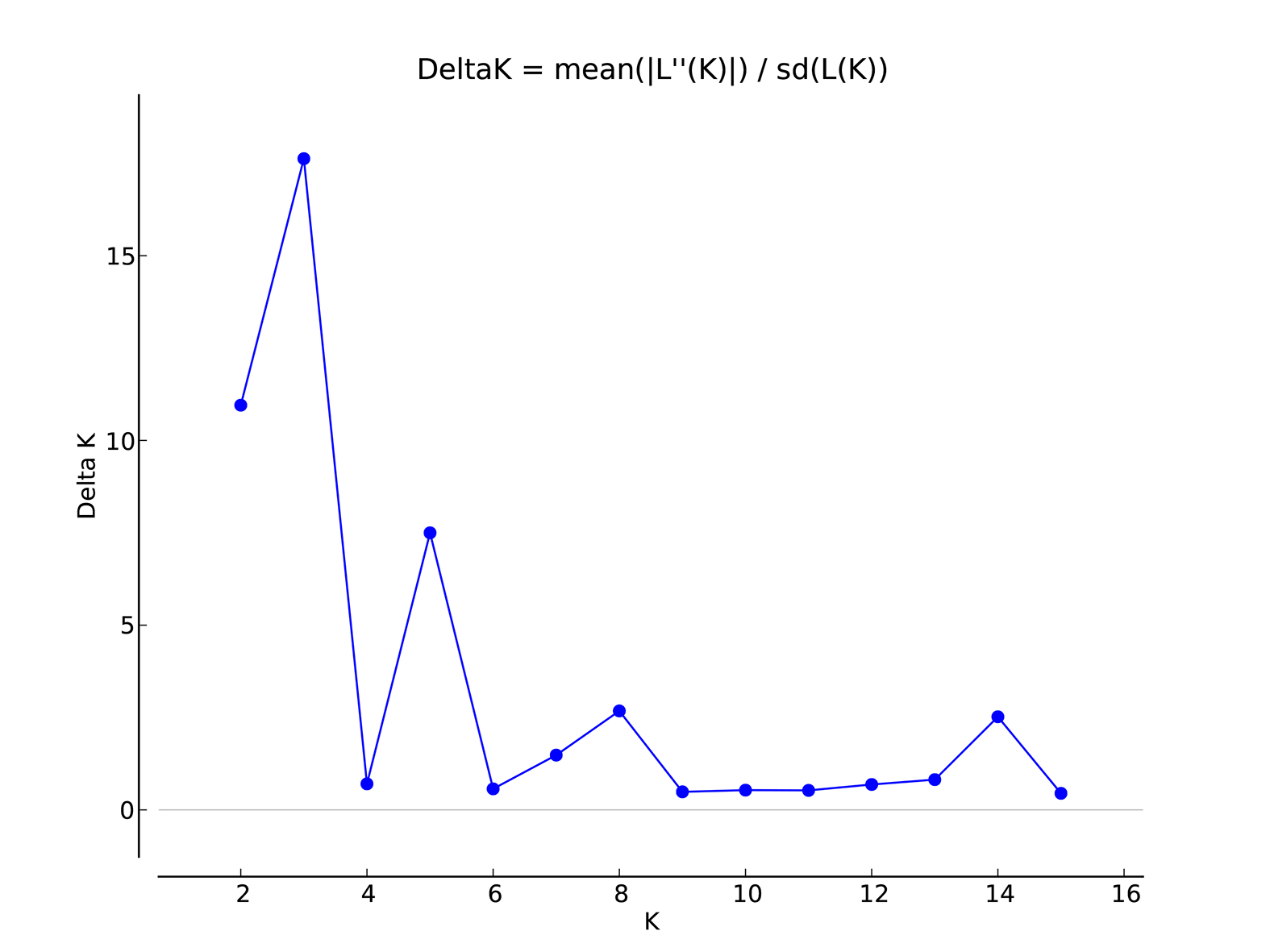

Supplement: S3 Fig — (TIF) [file pone.0154020.s003.tif]

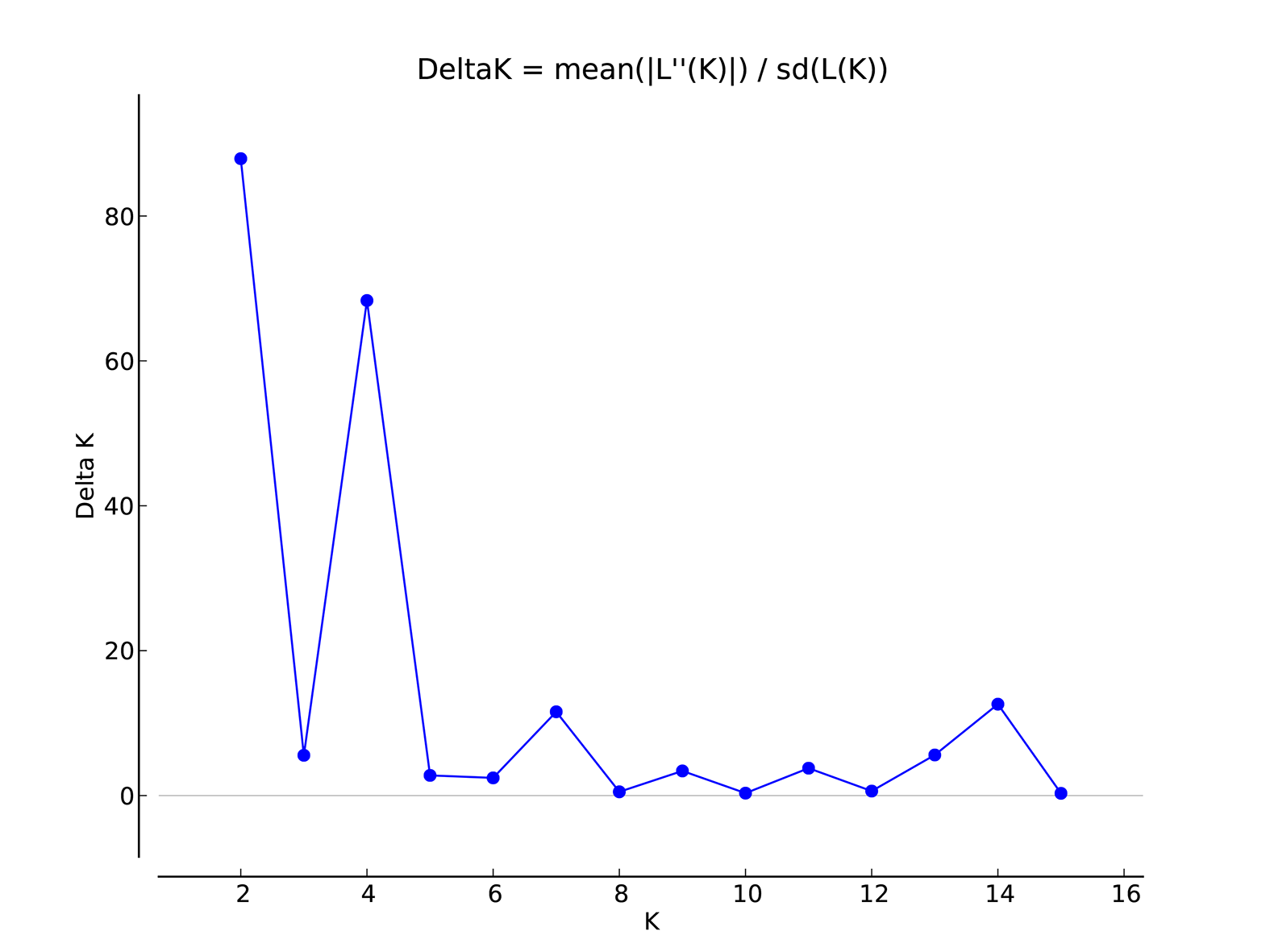

Supplement: S4 Fig — (TIF) [file pone.0154020.s004.tif]
