# Supplementary material for: Population Structure and Adaptive Divergence in a High Gene Flow Marine Fish: The Small Yellow Croaker (Larimichthys polyactis)
Source: PLoS One. 2016 Apr 21;11(4):e0154020. doi: 10.1371/journal.pone.0154020 (PMC4839715; doi:10.1371/journal.pone.0154020)
Supplement: S1 Table — (DOCX) [file pone.0154020.s006.docx]

### Table S1. Summary of the statistics for fifteen microsatellite loci

| Locus | Parameter | DD | BLA | BLB | QHD | DY | WH | QD | SYA | SYB | SYC | CJK | NEA | NEB | WL | XP |
| --- | --- | --- | --- | --- | --- | --- | --- | --- | --- | --- | --- | --- | --- | --- | --- | --- |
| Lpol02 | *Na* | 11 | 8 | 11 | 7 | 6 | 10 | 9 | 9 | 9 | 7 | 10 | 11 | 9 | 12 | 11 |
|  | *H*_E_ | 0.790 | 0.796 | 0.771 | 0.763 | 0.759 | 0.808 | 0.794 | 0.822 | 0.823 | 0.837 | 0.837 | 0.813 | 0.759 | 0.795 | 0.833 |
|  | *H*_O_ | 0.792 | 0.833 | 0.792 | 0.667 | 0.667 | 0.667 | 0.800 | 0.750 | 0.792 | 0.650 | 0.650 | 0.522 | 0.621 | 0.773 | 0.798 |
|  | *PIC* | 0.753 | 0.749 | 0.733 | 0.706 | 0.700 | 0.766 | 0.745 | 0.781 | 0.782 | 0.790 | 0.796 | 0.777 | 0.722 | 0.762 | 8.890 |
|  | *A*_R_ | 8.323 | 6.665 | 7.970 | 6.560 | 5.790 | 7.828 | 7.433 | 7.627 | 7.548 | 6.437 | 8.527 | 8.823 | 7.405 | 9.451 | 0.001 |
|  | *F_IS_* | -0.002 | -0.048 | -0.027 | 0.130 | 0.125 | 0.178 | -0.008 | 0.089 | 0.039 | 0.228 | 0.228 | 0.363 | 0.184 | 0.029 | 0.803 |
| Lpol03 | *Na* | 12 | 10 | 10 | 10 | 8 | 16 | 11 | 10 | 9 | 18 | 10 | 14 | 14 | 12 | 11 |
|  | *H*_E_ | 0.800 | 0.809 | 0.850 | 0.793 | 0.832 | 0.796 | 0.756 | 0.803 | 0.844 | 0.923 | 0.705 | 0.915 | 0.863 | 0.831 | 0.708 |
|  | *H*_O_ | 0.792 | 0.875 | 0.875 | 0.667 | 0.733 | 0.750 | 0.650 | 0.833 | 0.792 | 0.696 | 0.800 | 0.826 | 0.767 | 0.773 | 0.766 |
|  | *PIC* | 0.766 | 0.773 | 0.813 | 0.748 | 0.781 | 0.768 | 0.720 | 0.763 | 0.804 | 0.895 | 0.666 | 0.886 | 0.833 | 0.799 | 8.353 |
|  | *A*_R_ | 9.091 | 8.406 | 8.096 | 8.980 | 7.555 | 10.762 | 8.818 | 7.876 | 7.324 | 12.582 | 7.763 | 11.364 | 9.470 | 10.022 | 0.120 |
|  | *F_IS_* | 0.010 | -0.084 | -0.030 | 0.164 | 0.123 | 0.059 | 0.144 | -0.038 | 0.063 | 0.250 | -0.139 | 0.099 | 0.113 | 0.072 | 0.886 |
| Lpol04 | *Na* | 18 | 16 | 14 | 9 | 10 | 15 | 13 | 17 | 11 | 13 | 13 | 20 | 18 | 18 | 18 |
|  | *H*_E_ | 0.843 | 0.885 | 0.887 | 0.844 | 0.865 | 0.895 | 0.856 | 0.926 | 0.840 | 0.906 | 0.863 | 0.938 | 0.913 | 0.914 | 0.750 |
|  | *H*_O_ | 0.833 | 0.750 | 0.625 | 0.800 | **0.438** | 0.652 | 0.550 | 0.708 | 0.542 | 0.609 | 0.750 | 0.696 | 0.533 | 0.773 | 0.856 |
|  | *PIC* | 0.809 | 0.856 | 0.855 | 0.793 | 0.820 | 0.863 | 0.823 | 0.900 | 0.808 | 0.876 | 0.826 | 0.912 | 0.889 | 0.886 | 11.977 |
|  | *A*_R_ | 11.149 | 11.569 | 10.183 | 8.326 | 9.069 | 10.864 | 10.692 | 12.549 | 9.197 | 10.576 | 10.308 | 13.848 | 11.708 | 13.035 | 0.156 |
|  | *F_IS_* | 0.012 | 0.155 | 0.299 | 0.054 | 0.502 | 0.276 | 0.364 | 0.239 | 0.360 | 0.333 | 0.134 | 0.263 | 0.420 | 0.158 | 0.931 |
| Lpol05 | *Na* | 16 | 17 | 17 | 16 | 10 | 19 | 15 | 16 | 15 | 14 | 16 | 19 | 16 | 16 | 16 |
|  | *H*_E_ | 0.922 | 0.927 | 0.905 | 0.933 | 0.849 | 0.941 | 0.921 | 0.921 | 0.882 | 0.856 | 0.914 | 0.936 | 0.918 | 0.903 | 0.833 |
|  | *H*_O_ | 0.625 | 0.739 | 0.750 | 0.800 | 0.429 | 0.667 | 0.700 | 0.708 | 0.667 | **0.478** | 0.900 | 0.826 | 0.517 | 0.727 | 0.905 |
|  | *PIC* | 0.895 | 0.900 | 0.878 | 0.895 | 0.799 | 0.917 | 0.889 | 0.894 | 0.853 | 0.826 | 0.884 | 0.910 | 0.894 | 0.873 | 12.325 |
|  | *A*_R_ | 12.149 | 13.119 | 12.429 | 13.921 | 9.380 | 13.830 | 12.192 | 12.028 | 11.069 | 10.894 | 12.738 | 13.756 | 11.767 | 11.828 | 0.107 |
|  | *F_IS_* | 0.327 | 0.206 | 0.174 | 0.147 | 0.505 | 0.296 | 0.244 | 0.235 | 0.248 | 0.447 | 0.016 | 0.120 | 0.441 | 0.198 | 0.965 |
| Lpol06 | *Na* | 26 | 18 | 19 | 18 | 19 | 22 | 17 | 21 | 19 | 18 | 16 | 20 | 18 | 18 | 26 |
|  | *H*_E_ | 0.970 | 0.940 | 0.938 | 0.959 | 0.968 | 0.941 | 0.945 | 0.959 | 0.958 | 0.956 | 0.942 | 0.955 | 0.941 | 0.949 | 0.708 |
|  | *H*_O_ | 0.833 | 0.708 | 0.792 | 0.933 | 1.000 | 0.708 | 0.900 | **0.625** | 0.750 | 0.913 | 0.900 | 0.913 | 0.733 | 0.727 | 0.942 |
|  | *PIC* | 0.947 | 0.915 | 0.913 | 0.922 | 0.934 | 0.916 | 0.916 | 0.936 | 0.935 | 0.931 | 0.913 | 0.930 | 0.921 | 0.923 | 16.726 |
|  | *A*_R_ | 17.272 | 13.512 | 13.738 | 15.681 | 16.321 | 14.274 | 13.716 | 15.375 | 14.856 | 14.406 | 13.161 | 14.778 | 13.114 | 13.990 | 0.270 |
|  | *F_IS_* | 0.143 | 0.250 | 0.159 | 0.027 | -0.034 | 0.251 | 0.049 | 0.353 | 0.221 | 0.045 | 0.046 | 0.044 | 0.224 | 0.238 | 0.832 |
| Lpol08 | *Na* | 10 | 10 | 12 | 9 | 9 | 10 | 9 | 12 | 9 | 18 | 10 | 10 | 11 | 9 | 10 |
|  | *H*_E_ | 0.824 | 0.840 | 0.892 | 0.851 | 0.817 | 0.840 | 0.824 | 0.827 | 0.823 | 0.939 | 0.827 | 0.863 | 0.854 | 0.862 | 0.708 |
|  | *H*_O_ | 0.792 | 0.750 | 0.917 | 0.933 | 0.563 | 0.792 | 0.900 | 0.833 | 0.750 | 0.909 | 0.700 | 0.870 | 0.633 | 0.818 | 0.791 |
|  | *PIC* | 0.780 | 0.801 | 0.860 | 0.801 | 0.762 | 0.799 | 0.778 | 0.788 | 0.785 | 0.912 | 0.781 | 0.826 | 0.820 | 0.823 | 7.691 |
|  | *A*_R_ | 7.583 | 7.720 | 9.430 | 8.462 | 7.739 | 7.868 | 7.533 | 8.644 | 7.690 | 13.627 | 7.921 | 8.251 | 8.038 | 7.913 | 0.151 |
|  | *F_IS_* | 0.040 | 0.110 | -0.028 | -0.101 | 0.318 | 0.058 | -0.094 | -0.008 | 0.090 | 0.032 | 0.157 | -0.008 | 0.261 | 0.051 | 0.966 |
| Lpol09 | *Na* | 25 | 19 | 19 | 16 | 16 | 20 | 18 | 20 | 19 | 17 | 20 | 22 | 21 | 20 | 22 |
|  | *H*_E_ | 0.969 | 0.949 | 0.947 | 0.910 | 0.950 | 0.943 | 0.957 | 0.954 | 0.933 | 0.946 | 0.954 | 0.960 | 0.952 | 0.936 | 0.667 |
|  | *H*_O_ | 0.792 | 0.792 | 0.667 | 0.867 | 0.625 | 0.522 | 0.737 | 0.833 | **0.625** | 0.818 | 0.700 | 1.000 | 0.690 | 0.818 | 0.943 |
|  | *PIC* | 0.946 | 0.924 | 0.922 | 0.871 | 0.914 | 0.918 | 0.928 | 0.930 | 0.908 | 0.920 | 0.926 | 0.936 | 0.931 | 0.909 | 16.231 |
|  | *A*_R_ | 17.123 | 14.425 | 13.905 | 13.726 | 13.986 | 14.378 | 14.813 | 14.856 | 13.827 | 13.565 | 15.108 | 15.606 | 14.477 | 14.321 | 0.315 |
|  | *F_IS_* | 0.186 | 0.168 | 0.300 | 0.050 | 0.349 | 0.452 | 0.235 | 0.129 | 0.335 | 0.138 | 0.271 | -0.042 | 0.279 | 0.128 | 0.851 |
| Lpol10 | *Na* | 14 | 13 | 13 | 9 | 8 | 14 | 11 | 13 | 14 | 12 | 8 | 12 | 13 | 10 | 13 |
|  | *H*_E_ | 0.908 | 0.865 | 0.874 | 0.864 | 0.788 | 0.876 | 0.806 | 0.888 | 0.874 | 0.816 | 0.813 | 0.836 | 0.859 | 0.831 | 0.833 |
|  | *H*_O_ | 1.000 | 0.792 | 0.875 | 0.800 | 0.813 | 0.833 | 0.900 | 0.833 | 0.583 | 0.783 | 0.900 | 0.609 | 0.733 | 0.818 | 0.814 |
|  | *PIC* | 0.879 | 0.831 | 0.841 | 0.815 | 0.728 | 0.843 | 0.763 | 0.857 | 0.843 | 0.782 | 0.764 | 0.800 | 0.829 | 0.792 | 9.402 |
|  | *A*_R_ | 10.834 | 9.782 | 9.633 | 8.195 | 6.944 | 9.933 | 8.676 | 9.840 | 10.292 | 9.244 | 6.967 | 9.251 | 9.448 | 8.224 | 0.021 |
|  | *F_IS_* | -0.104 | 0.087 | -0.001 | 0.077 | -0.032 | 0.050 | -0.119 | 0.063 | 0.337 | 0.042 | -0.110 | 0.276 | 0.148 | 0.016 | 0.811 |
| Lpol11 | *Na* | 7 | 8 | 7 | 7 | 5 | 7 | 8 | 8 | 7 | 8 | 7 | 6 | 8 | 6 | 9 |
|  | *H*_E_ | 0.794 | 0.755 | 0.797 | 0.747 | 0.692 | 0.777 | 0.815 | 0.793 | 0.761 | 0.802 | 0.774 | 0.752 | 0.773 | 0.781 | 0.708 |
|  | *H*_O_ | 0.667 | 0.750 | 0.708 | 0.733 | 0.375 | 0.708 | 0.750 | 0.625 | 0.917 | 0.783 | 0.500 | 0.696 | 0.700 | 0.818 | 0.768 |
|  | *PIC* | 0.747 | 0.710 | 0.751 | 0.684 | 0.612 | 0.728 | 0.766 | 0.746 | 0.702 | 0.759 | 0.720 | 0.689 | 0.723 | 0.725 | 7.402 |
|  | *A*_R_ | 6.273 | 6.814 | 6.322 | 6.365 | 4.692 | 6.165 | 6.885 | 6.834 | 5.638 | 7.003 | 6.235 | 4.999 | 6.114 | 5.339 | 0.129 |
|  | *F_IS_* | 0.164 | 0.007 | 0.113 | 0.019 | 0.466 | 0.091 | 0.082 | 0.215 | -0.211 | 0.025 | 0.360 | 0.076 | 0.096 | -0.049 | 0.910 |
| Lpol12 | *Na* | 18 | 19 | 19 | 16 | 13 | 18 | 15 | 16 | 19 | 17 | 15 | 20 | 21 | 18 | 18 |
|  | *H*_E_ | 0.930 | 0.949 | 0.944 | 0.938 | 0.923 | 0.923 | 0.874 | 0.937 | 0.943 | 0.906 | 0.879 | 0.949 | 0.948 | 0.938 | 0.792 |
|  | *H*_O_ | 0.833 | 0.875 | 0.917 | 0.933 | 0.875 | 0.875 | 0.800 | 0.917 | 0.833 | 1.000 | 0.850 | 0.913 | 0.867 | 0.909 | 0.883 |
|  | *PIC* | 0.904 | 0.925 | 0.919 | 0.900 | 0.885 | 0.896 | 0.840 | 0.912 | 0.919 | 0.877 | 0.846 | 0.924 | 0.928 | 0.910 | 12.278 |
|  | *A*_R_ | 13.049 | 14.321 | 13.680 | 14.082 | 11.599 | 12.542 | 11.124 | 12.746 | 14.268 | 11.894 | 11.506 | 14.370 | 14.430 | 13.291 | 0.133 |
|  | *F_IS_* | 0.106 | 0.080 | 0.030 | 0.005 | 0.054 | 0.053 | 0.087 | 0.022 | 0.119 | -0.106 | 0.034 | 0.039 | 0.087 | 0.031 | 0.909 |
| Lpol13 | *Na* | 15 | 13 | 15 | 11 | 10 | 15 | 15 | 16 | 14 | 12 | 14 | 12 | 15 | 15 | 15 |
|  | *H*_E_ | 0.894 | 0.864 | 0.880 | 0.874 | 0.841 | 0.892 | 0.912 | 0.918 | 0.861 | 0.893 | 0.856 | 0.873 | 0.871 | 0.892 | 0.917 |
|  | *H*_O_ | 0.875 | 0.875 | 0.875 | 1.000 | 0.750 | 0.917 | 0.900 | 0.875 | 0.792 | 0.783 | 0.950 | 0.870 | 0.967 | 1.000 | 0.880 |
|  | *PIC* | 0.865 | 0.831 | 0.853 | 0.829 | 0.795 | 0.862 | 0.879 | 0.891 | 0.831 | 0.862 | 0.823 | 0.841 | 0.843 | 0.862 | 11.316 |
|  | *A*_R_ | 11.245 | 9.843 | 11.572 | 10.087 | 9.058 | 11.115 | 11.738 | 11.922 | 10.667 | 10.290 | 11.111 | 9.708 | 10.060 | 11.531 | -0.009 |
|  | *F_IS_* | 0.021 | -0.013 | 0.006 | -0.151 | 0.111 | -0.028 | 0.013 | 0.048 | 0.082 | 0.126 | -0.112 | 0.005 | -0.112 | -0.124 | 0.899 |
| Lpol14 | *Na* | 12 | 14 | 15 | 9 | 12 | 11 | 10 | 15 | 14 | 12 | 16 | 15 | 12 | 15 | 16 |
|  | *H*_E_ | 0.882 | 0.882 | 0.899 | 0.871 | 0.899 | 0.874 | 0.878 | 0.892 | 0.911 | 0.863 | 0.918 | 0.890 | 0.867 | 0.908 | 0.917 |
|  | *H*_O_ | 0.792 | 0.750 | 1.000 | 0.867 | 0.813 | 0.917 | 0.850 | 0.708 | 0.792 | 1.000 | 0.900 | 1.000 | 0.800 | 0.773 | 0.870 |
|  | *PIC* | 0.849 | 0.851 | 0.869 | 0.824 | 0.858 | 0.840 | 0.840 | 0.862 | 0.883 | 0.829 | 0.887 | 0.859 | 0.838 | 0.878 | 11.575 |
|  | *A*_R_ | 9.479 | 10.661 | 11.292 | 8.389 | 10.430 | 8.757 | 8.774 | 10.926 | 10.878 | 9.459 | 12.630 | 10.992 | 9.425 | 11.396 | -0.020 |
|  | *F_IS_* | 0.105 | 0.153 | -0.115 | 0.005 | 0.099 | -0.050 | 0.033 | 0.209 | 0.134 | -0.163 | 0.020 | -0.127 | 0.078 | 0.152 | 0.946 |
| Lpol15 | *Na* | 24 | 21 | 21 | 18 | 18 | 22 | 22 | 21 | 24 | 19 | 23 | 21 | 27 | 21 | 24 |
|  | *H*_E_ | 0.966 | 0.957 | 0.949 | 0.966 | 0.956 | 0.955 | 0.964 | 0.923 | 0.958 | 0.938 | 0.965 | 0.961 | 0.961 | 0.959 | 0.875 |
|  | *H*_O_ | 0.917 | 0.917 | 0.875 | 0.933 | 0.813 | 0.958 | 0.950 | 0.833 | 0.875 | 0.870 | 1.000 | 1.000 | 0.833 | 0.909 | 0.922 |
|  | *PIC* | 0.944 | 0.933 | 0.925 | 0.929 | 0.921 | 0.931 | 0.937 | 0.897 | 0.935 | 0.912 | 0.938 | 0.937 | 0.942 | 0.933 | 15.674 |
|  | *A*_R_ | 16.528 | 15.212 | 14.522 | 15.978 | 15.388 | 15.281 | 16.532 | 13.928 | 15.818 | 13.647 | 16.672 | 15.538 | 16.319 | 15.475 | 0.076 |
|  | *F_IS_* | 0.052 | 0.043 | 0.080 | 0.034 | 0.154 | -0.004 | 0.015 | 0.099 | 0.089 | 0.075 | -0.037 | -0.041 | 0.135 | 0.053 | 0.837 |
| Lpol16 | *Na* | 16 | 16 | 14 | 13 | 15 | 16 | 13 | 11 | 14 | 17 | 16 | 17 | 20 | 15 | 14 |
|  | *H*_E_ | 0.912 | 0.923 | 0.875 | 0.853 | 0.873 | 0.926 | 0.892 | 0.886 | 0.878 | 0.907 | 0.923 | 0.920 | 0.898 | 0.912 | 0.708 |
|  | *H*_O_ | 0.667 | 0.667 | 0.667 | 0.667 | 0.750 | 0.792 | 0.700 | 0.583 | 0.708 | 0.783 | **0.650** | 0.739 | 0.667 | 0.864 | **0.799** |
|  | *PIC* | 0.884 | 0.896 | 0.843 | 0.812 | 0.833 | 0.899 | 0.857 | 0.854 | 0.846 | 0.879 | 0.892 | 0.892 | 0.873 | 0.882 | 9.264 |
|  | *A*_R_ | 11.749 | 12.246 | 10.253 | 11.487 | 12.331 | 12.209 | 10.459 | 9.276 | 10.579 | 12.342 | 12.429 | 12.578 | 12.049 | 11.700 | 0.156 |
|  | *F_IS_* | 0.273 | 0.282 | 0.242 | 0.224 | 0.145 | 0.147 | 0.220 | 0.346 | 0.196 | 0.140 | 0.301 | 0.200 | 0.261 | 0.055 | 0.922 |
| Lpol17 | *Na* | 19 | 20 | 21 | 16 | 11 | 19 | 19 | 19 | 17 | 19 | 19 | 18 | 18 | 21 | 20 |
|  | *H*_E_ | 0.918 | 0.948 | 0.946 | 0.931 | 0.920 | 0.947 | 0.951 | 0.911 | 0.919 | 0.939 | 0.953 | 0.944 | 0.935 | 0.932 | 0.792 |
|  | *H*_O_ | 0.875 | 0.833 | 0.708 | 0.733 | 0.667 | 0.792 | 0.850 | 0.833 | **0.609** | 0.826 | 0.800 | 0.870 | 0.700 | 0.909 | 0.922 |
|  | *PIC* | 0.891 | 0.923 | 0.922 | 0.892 | 0.870 | 0.922 | 0.923 | 0.884 | 0.891 | 0.913 | 0.924 | 0.918 | 0.914 | 0.905 | 0.896 |
|  | *A*_R_ | 13.304 | 14.196 | 14.790 | 13.895 | 11.000 | 13.951 | 14.605 | 12.635 | 12.577 | 13.534 | 14.791 | 13.505 | 12.835 | 14.426 | 13.620 |
|  | *F_IS_* | 0.047 | 0.123 | 0.255 | 0.218 | 0.285 | 0.167 | 0.109 | 0.087 | 0.343 | 0.123 | 0.164 | 0.080 | 0.255 | 0.026 | 0.144 |
|  | Average |  |  |  |  |  |  |  |  |  |  |  |  |  |  |  |
|  | *H*_E_ | 0.888 | 0.886 | 0.890 | 0.873 | 0.862 | 0.889 | 0.876 | 0.891 | 0.881 | 0.895 | 0.875 | 0.900 | 0.887 | 0.889 | 0.887 |
|  | *H*_O_ | 0.891 | 0.923 | 0.922 | 0.892 | 0.870 | 0.922 | 0.923 | 0.884 | 0.891 | 0.913 | 0.924 | 0.918 | 0.914 | 0.905 | 0.896 |
|  | *PIC* | 0.857 | 0.855 | 0.860 | 0.828 | 0.814 | 0.858 | 0.840 | 0.860 | 0.848 | 0.864 | 0.839 | 0.869 | 0.860 | 0.857 | 11.515 |
|  | *A*_R_ | 11.677 | 11.233 | 11.188 | 10.942 | 10.085 | 11.317 | 10.933 | 11.137 | 10.815 | 11.300 | 11.191 | 11.824 | 11.111 | 11.463 | 0.856 |
| All | *F*_IS_ | 0.095 | 0.106 | 0.100 | 0.060 | 0.209 | 0.136 | 0.094 | 0.142 | 0.168 | 0.116 | 0.092 | 0.087 | 0.194 | 0.072 | 0.119 |

Number of alleles (*N*a), observed heterozygosity (*H*_O_), expected heterozygosity (*H*_E_), Polymorphism Information Content (*PIC*), allelic richness (*A*_R_) and inbreeding coefficient (*F*_IS_). Bold type indicates significant deviations from Hardy-Weinberg equilibrium after Bonferroni correction (*P* < 0.0005).
