# Supplementary material for: Population Structure and Adaptive Divergence in a High Gene Flow Marine Fish: The Small Yellow Croaker (Larimichthys polyactis)
Source: PLoS One. 2016 Apr 21;11(4):e0154020. doi: 10.1371/journal.pone.0154020 (PMC4839715; doi:10.1371/journal.pone.0154020)
Supplement: S2 Table — (DOCX) [file pone.0154020.s007.docx]

### S2 Table. Analysis of molecular variance (AMOVA) based on neutral microsatellite datasets

| Source of variation | Sum of squares | Variance components | Percentage variation | | *F-*statistics | | *P* values |
| --- | --- | --- | --- | --- | --- | --- | --- |
| One group |  |  |  | |  | |  |
| Among populations | 112.324 | 0.022 | 0.356 | | *F*_ST_=0.0036 | | 1.000 |
| Among individuals within populations | 2252.010 | 0.778 | 12.412 | | *F*_IS_=0.125 | | **0.000** |
| Within individuals | 1836.000 | 5.470 | 87.231 | | *F*_IT_=0.128 | | **0.000** |
| **Cluster according to geographic sampling range** |  |  |  | |  | |  |
| Two groups（DD,BLA,BLB,QHD,DY,WH,QD）（SYA,SYB,SYC,CJK,NEA,NEB,WL,XP） | | |  | |  | |  |
| Among groups | 9.135 | 0.004 | 0.057 | | *F*_CT_=0.0006 | | 0.112 |
| Among populations within groups | 103.189 | 0.020 | 0.326 | | *F*_SC_=0.0033 | | **0.000** |
| Among individuals within populations | 2252.010 | 0.778 | 12.409 | | *F*_IS_ =0.125 | | **0.000** |
| Within individuals | 1836.000 | 5.470 | 87.208 | | *F*_IT_ =0.128 | | **0.000** |
| Four groups（DD,BLA,BLB,QHD）（DY,WH,QD）（SYA,SYB,SYC,CJK）（NEA,NEB,WL,XP） | | |  |  | |  | |
| Among groups | 25.658 | 0.004 | 0.064 | | *F*_CT_ =0.0006 | | 0.131 |
| Among populations within groups | 86.667 | 0.019 | 0.305 | | *F*_SC_ =0.003 | | **0.0009** |
| Among individuals within populations | 2252.010 | 0.778 | 12.411 | | *F*_IS_ =0.125 | | **0.000** |
| Within individuals | 1836.000 | 5.470 | 87.220 | | *F*_IT_ =0.128 | | **0.000** |
| **Cluster according to sampling time** |  |  |  | |  | |  |
| Two groups（DD,QHD, CJK, WL,XP）（BLA,BLB, DY,WH,QD,SYA,SYB,SYC, NEA,NEB,） | | |  |  | |  | |
| Among groups | 12.172 | 0.001 | 0.248 | | *F*_CT_=0.0025 | | **0.000** |
| Among populations within groups | 100.152 | 0.063 | 0.242 | | *F*_SC_=0.0024 | | **0.003** |
| Among individuals within populations | 2252.010 | 0.778 | 12.400 | | *F*_IS_ =0.125 | | **0.000** |
| Within individuals | 1836.000 | 5.470 | 87.116 | | *F*_IT_ =0.128 | | **0.000** |
| Four groups（SYA,SYB,SYC）（BLA,BLB, NEA,NEB,QHD）（DY,WH,QD）（DD,QHD, CJK, WL,XP） | | |  | |  | |  |
| Among groups | 29.319 | 0.012 | 0.215 | | *F*_CT_ =0.0022 | | **0.000** |
| Among populations within groups | 83.005 | 0.054 | 0.186 | | *F*_SC_ =0.0019 | | **0.020** |
| Among individuals within populations | 2252.010 | 0.778 | 12.407 | | *F*_IS_ =0.125 | | **0.000** |
| Within individuals | 1836.000 | 5.470 | 87.192 | | *F*_IT_ =0.128 | | **0.000** |

Bold *P* numbers are significant values. Refer to Table 1 for abbreviations of sampling sites
