# Supplementary material for: Population Structure and Adaptive Divergence in a High Gene Flow Marine Fish: The Small Yellow Croaker (Larimichthys polyactis)
Source: PLoS One. 2016 Apr 21;11(4):e0154020. doi: 10.1371/journal.pone.0154020 (PMC4839715; doi:10.1371/journal.pone.0154020)
Supplement: S3 Table — (DOCX) [file pone.0154020.s008.docx]

### S3 Table. Signed rank Wilcoxon test of the mutation–drift equilibrium estimated for fifteen microsatellite loci in populations of *L. polyactis.*

| Populations | Wilcoxon signed-rank test | | | Mode shift |
| --- | --- | --- | --- | --- |
|  | IAM | TPM90 | SMM |  |
| DD | **0.03186** | 0.59802 | 0.99097 | Yes |
| BLA | **0.00021** | 0.40198 | 0.98721 | Yes |
| BLB | **0.00134** | 0.70026 | 0.99866 | Yes |
| QHD | 0.17957 | 0.84860 | 0.99893 | Yes |
| DY | **0.00513** | 0.24435 | 0.94650 | Yes |
| WH | **0.00418** | 0.80530 | 0.99971 | Yes |
| QD | **0.03650** | 0.64014 | 0.97232 | Yes |
| SYA | **0.00066** | 0.31934 | 0.96350 | Yes |
| SYB | **0.00008** | 0.53296 | 0.99832 | Yes |
| SYC | **0.00066** | 0.42346 | 0.95837 | Yes |
| CJK | 0.07571 | 0.77286 | 0.99893 | Yes |
| NEA | **0.00011** | 0.15140 | 0.90619 | Yes |
| NEB | **0.00029** | 0.48898 | 0.99097 | Yes |
| WL | **0.03186** | 0.73776 | 0.99832 | Yes |
| XP | 0.21060 | 0.96814 | 0.99971 | Yes |

The infinite alleles model (IAM), a two-phase model (TPM90) for a 10% occurrence of multiple steps, and the stepwise mutation model (SMM).

Significant values (*P* < 0.05) are highlighted in bold.

Yes: normal L-shaped distribution; No: shifted mode.
